# Influence of Physical Exercise on Psychological Well-Being of Young Adults: A Quantitative Study

**DOI:** 10.3390/ijerph19074282

**Published:** 2022-04-03

**Authors:** Jesús Granero-Jiménez, María Mar López-Rodríguez, Iria Dobarrio-Sanz, Alda Elena Cortés-Rodríguez

**Affiliations:** 1 Observation Unit, Emergency Department, Torrecárdenas University Hospital, 04009 Almería, Spain; jgj235@inlumine.ual.es; 2 Department of Nursing, Physiotherapy and Medicine, University of Almería, 04120 Almería, Spain; ids135@ual.es (I.D.-S.); cra566@ual.es (A.E.C.-R.)

**Keywords:** exercise, psychological well-being, young adults, motivation, intrinsic motivation

## Abstract

Physical activity is a key element in people’s health as it provides important physical benefits, as well as improves mental health and quality of life. However, recent years have seen an increase in the percentage of young adults showing high levels of inactivity. Although, it has been observed that the motivation to perform physical activity seems to be an important factor when starting and then keeping it up. Thus, the general aim of this work was to explore the association between physical activity, motivation, and psychological well-being in young adults. To do this, a descriptive cross-sectional correlational study was carried out together with a multiple linear regression analysis. An online survey was applied between December 2017 and the first quarter of 2018, in which the level of activity, motivation, and psychological well-being of the participants were measured. Starting from a final sample of 489 subjects aged between 18 and 35 years, a higher level of physical activity was found to be related to higher psychological well-being. In addition, motivation, and more specifically intrinsic motivation, was an important determinant of psychological well-being, gaining greater influence among male participants who had a higher level of physical activity. This study therefore emphasizes the clear influence of physical activity on the psychological well-being of young adults and highlights the need to work on intrinsic motivation to improve levels of physical activity.

## 1. Introduction

Lack of physical activity is one of the main risk factors for premature death, along with smoking and poor diet [1,2,3]. It is recommended that young adults engage in at least an average of 60 min per day of moderate physical activity or a minimum of 150 min of vigorous physical activity per week [2]. Despite this, globally, physical inactivity and a sedentary lifestyle occur in almost 30% of adults and in more than 80% of school-age adolescents [2]. In general, the population is more physically inactive in developed countries [1,4,5,6]. Likewise, there are also differences between the sexes, with girls and young women being the most sedentary [5,6]. In addition, it is known that during the passage from adolescence to adulthood there is a greater decrease in physical activity [5,7,8,9], this decrease coinciding with the beginning of university study [10]. This situation has now been worsened by the COVID-19 pandemic [11].

Physical activity implies multiple benefits, such as the prevention of cardiovascular diseases [12], better control of body weight [10,13], less presence of chronic pain [14], better bone density and better physical fitness [15,16], greater muscle strength, and lower risk of mortality [13,17,18]. In addition, at a psychological level, physical activity improves mental health and quality of life [8,19], reduces stress and anxiety [8,20,21], lowers the risk of depression [22], and improves the quality of sleep [23]. Likewise, physical activity is positively related to better academic performance among university students [24]. In short, physical activity and exercise are associated with psychological and social well-being [25].

Psychological well-being is made up of self-acceptance, positive relationships, autonomy, mastery of the environment, purpose in life, and personal growth. [26]. It is known that these benefits are affected by the prior beliefs and expectations that the person had towards that physical activity [27]. Similarly, there are a number of circumstances that make exercise difficult or prevent it, such as lack of time [28], lack of social support [29], economic difficulties [29], physical discomfort [30], and lack of motivation [29,30,31,32]. Specifically, research has found that one of the factors that most influences physical activity performance is self-determined motivation [33,34,35,36,37,38].

According to Self-Determination Theory (SDT) [38,39,40], motivation occurs as a continuous process involving different degrees of autonomy [40]. SDT establishes two types of motivation: intrinsic, which refers to more self-determined behaviours where the person acts of their own free will or for their own enjoyment, and extrinsic, which refers to more controlled behaviours where the individual’s motivation comes from the outside and implies a certain pressure to behave in a certain way [35,36,37,38,39,40]. Thus, if a person gets involved in an activity out of interest in the activity itself, it will be easier for them to adhere to this activity; whereas, if the person performs an activity for instrumental reasons or to obtain certain results, such as rewards or social recognition, it will be more difficult for them to reach an adequate level of involvement and for the behaviour to be consolidated [33,37,38,39].

SDT also tells us that the satisfaction of basic psychological needs (competence, autonomy, and relationships with others) can promote self-determined motivation and, in turn, have repercussions on the cognitive, affective, and behavioural consequences of psychosocial well-being [37,39,40]. In addition, those social contexts that satisfy people’s competence, autonomy, and relationships will also foster intrinsic motivation and, therefore, more autonomous and lasting behaviours [35,39,40]. Given that SDT seems to be a key in predicting self-determined behaviors related to physical activity among university students, and that there are few studies on this topic and its relationship with psychological and social well-being in this population [36,41,42,43], this study was carried out. The studies carried out to date have investigated the quality of life [36]; they have focused only on men [41]; have studied life satisfaction [42]; or have wanted to develop a predictive model that relates physical activity and psychological aspects [43]. The aim of this study was thus to explore the association between physical exercise, motivation, and psychological well-being in young adults. More specifically, we tried to define those factors that motivate taking physical exercise and their influence on psychological well-being.

## 2. Materials and Methods

### 2.1. Study Design

A cross-sectional design was used, and the study was carried out between December 2017 and March 2018.

### 2.2. Settings and Participants

A convenience sampling method was used to recruit young adults from a city in southern Spain. The subjects were recruited through social media and the nursing faculty of the local university. The inclusion criteria were being between the ages of 18 and 35 years old and being Spanish speakers. The exclusion criteria were not being able to answer the survey due to any physical condition or not completing all the applied instruments.

### 2.3. Instruments

The demographic data collected were: age, sex, educational level (categorized into: primary education; lower secondary education; upper secondary education; no education), weight, height, Body Mass Index (BMI) (categorized into: underweight (<18.5); healthy (18.5–24.9); overweight (25.0–29.9); obese (>30)), taking exercise (categorized into: yes or no), and people who influence participants to exercise (categorized into: friends; family; partners; coworkers).

Three dependent variables were studied: physical exercise, psychological well-being, and motivation. Physical exercise was defined as any movement performed by skeletal muscles that require energy. This variable was assessed by the Spanish version of the International Physical Activity Questionnaire (IPAQ) short-version, which measures the physical activity performed in the last seven days using the Metabolic Equivalent of Task (MET) [44]. This short-version provides information about three types of physical activity: walking (i.e., During the last 7 days, on how many days did you walk for at least 10 min at a time?); moderate-intensity activities (i.e., During the last 7 days, on how many days did you do moderate physical activities like carrying light loads, bicycling at a regular pace, or doubles tennis?); and vigorous-intensity activities (i.e., During the last 7 days, on how many days did you do vigorous physical activities like heavy lifting, digging, aerobics, or fast bicycling?). The physical activity was conceptualized as a continuous variable, and it was categorized into a low level of physical activity (<600 METs minutes/week), moderate level of physical activity (600–1500 METs minutes/week), and high level of physical activity (>1500 METs minutes/week) [44]. The Spanish short-version of IPAQ was assessed on a Spanish population and showed a good reliability coefficient for total physical activity (r = 0.82) [45]

Psychological well-being was conceived as a subjective assessment that indicates life satisfaction as well as good quality of life. The Spanish adaptation of the Ryff Psychological Well-Being Scales [26] was used to evaluate this variable. This instrument consists of 39 items divided into 6 subscales: Self-acceptance (i.e., When I look at the story of my life, I am pleased with how things have turned out); Positive relations (i.e., People would describe me as a giving person, willing to share my time with others); Autonomy (i.e., I have confidence in my opinions, even if they are contrary to the general consensus); Environmental mastery (i.e., In general, I feel I am in charge of the situation in which I live); Purpose in life (i.e., Some people wander aimlessly through life, but I am not one of them); and Personal growth (i.e., I think it is important to have new experiences that challenge how you think about yourself and the world). These items were rated using a 6-point scale ranging from 1 (I totally disagree) to 6 (I totally agree). The overall psychological well-being was calculated by reverse-coding 21 items and obtaining the mean score of all 39 items, so that higher scores indicate greater psychological well-being. Moreover, separate subscale scores were calculated using the mean score of all items within each subscale. Regarding internal consistency, all the subscales of the Spanish adaptation of the Ryff Psychological Well-Being Scales were evaluated in a Spanish population and showed good internal consistency, with values between 0.71 and 0.83 [26].

Motivation was conceptualized as an individual drive that allows the subject to perform different behaviours to achieve an objective. In order to evaluate motivation in exercising, the Spanish adaptation of the Motivation for Physical Activity Measure-Revised (MPAM-R) [46] was applied. This scale is made up of 30 items divided into 5 subscales: Enjoyment (i.e., Because it makes me happy); Competence (i.e., Because I like physical challenges); Appearance (i.e., Because I want to lose weight, look better); Fitness (i.e., Because I want to improve cardiovascular fitness); and Social (i.e., Because I want to be with others in activity), but these subscales can be grouped into intrinsic motivation (including the Enjoyment and Competence subscales) and extrinsic motivation (including the Social, Fitness, and Appearance subscales). All items are measured on a 7-point scale ranging from 1 (Not at all true for me) and 7 (Very true for me). Both the general motivation score and the five subscales’ scores were calculated by obtaining the mean value of the corresponding items, so that a higher score means that the person has a higher motivation. The Spanish version of the MPAM-R demonstrated adequate reliability (α = 0.80 (Fitness); α = 0.81 (Social); α = 0.84 (Enjoyment); α = 0.85 (Competence); α = 0.87 (Appearance)) and validity on a sample of Spanish participants [46].

### 2.4. Data Analysis

Data analysis was performed using IBM SPSS Statistics^®^ for Windows, Version 25.0, Armonk, NY: IBM Corp USA. A descriptive analysis of sociodemographic variables was carried out using central tendency and dispersion measures and mean and standard deviation for quantitative variables and frequency and percentage measures for qualitative variables. A normal distribution of the data was verified using the Kolmogorov–Smirnov test. Since the data did not follow a normal distribution, the Mann–Whitney test and Kruskal–Wallis U test were used to compare the continuous data and chi-squared tests for the categorial data. An Exact Fisher test was calculated for ‘level of education’. In order to study the relationship between the quantitative variables, a Spearman’s correlation test was used. Finally, a multiple regression analysis was performed. For all the analyses, a *p*-value < 0.05 was considered to be statistically significant.

### 2.5. Ethical Considerations

All subjects gave their informed consent for inclusion before they participated in the study. The study was conducted in accordance with the Declaration of Helsinki, and the protocol was approved by the Ethics Committee of the Department of Nursing, Physiotherapy, and Medicine (EFM-15/19). Written detailed information was provided to all eligible subjects in order to inform them of the study’s aims and procedures.

Furthermore, participants were asked to voluntarily sign an informed consent document before participating, confirming that they understood the information provided and their right to withdraw from the study at any time without consequences.

All data were processed in accordance with the European Data Protection Legislation, governed by Regulation (EU) 2016/679 of the European Parliament and of the Council of 27 April 2016.

## 3. Results

### 3.1. Sample Characteristics

Table 1 shows the main demographic characteristics of the participants. The sample comprised 67.48% (*n* = 330) female participants with a mean age of 22.42 ± 3.27 years old. In addition, 57.24% of the participants (*n* = 280) had completed upper secondary education and 55.83% (*n* = 273) reported doing exercise. When they were asked about the people who most influenced them to exercise, 48.90% (*n* = 239) mentioned friends followed by family at 34.40% (*n* = 168). Finally, regarding BMI, the participants average was 23.63 ± 4.56 with 69.73% (*n* = 341) having a healthy BMI.

### 3.2. Outcome Measures

The results from the studied variables are shown in Table 2. In reference to physical activity, 44.17% (*n* = 216) were classified on a high level with the IPAQ mean score 3701.39 ± 3954.06. Moreover, participants’ average psychological well-being score was 4.47 ± 0.69, and the MPAM-R mean score was 3.39 ± 0.76.

### 3.3. Comparison of Groups

A comparison analysis of group differences according to physical activity is collected in Table 3. According to IPAQ results, no significant differences were observed in age, BMI, and educational level. However, there were significant differences in gender (X^2^ = 8.44; *p* = 0.02), as more male participants had a high level of physical activity. Another point of interest was that almost the same percentage of women participated in each level of physical activity while men mainly practiced a high or a low level of exercise.

On the other hand, there were significant differences between groups in psychological well-being and motivation. Those participants who had a low level of physical activity presented a low score in psychological well-being, intrinsic motivation, and extrinsic motivation. However, those who were classified with a high level of physical activity gained the highest scores in these three variables (See Table 3).

More specifically, there were significant differences according to gender in BMI (z = −4.93; *p* < 0.001), with male participants presenting higher scores. Moreover, men also obtained higher scores in general physical activity (z = −3.62; *p* = <.001), moderate-intensity activities (z = −3.23; *p* < 0.001), and vigorous-intensity activities (z = −4.36; *p* < 0.001), with a significant difference compared to women. Finally, significant differences were found in general motivation (z = −2.48; *p* = 0.01) and the subscale for intrinsic motivation (z = −2.99; *p* = 0.003), in which male participants obtained higher scores (Table 4).

### 3.4. Correlation Analysis

Table 5 shows the correlation analysis between the main studied variables. Regarding motivation, the IPAQ score was significantly correlated with all subscales of the MPAM-R in a moderate and positive way, excluding Social (r = 0.19; *p* < 0.001) and Appearance (r = 0.13; *p* < 0.001), which were variables with a weak correlation. In addition, the IPAQ score presented a positive and weak correlation with the Ryff Psychological Well-Being Scales (r = 0.10; *p* = 0.03) and the subscales for personal growth (r = 0.11; *p* = 0.01) and purpose in life (r = 0.11; *p* = 0.01). BMI also presented a weak correlation with the IPAQ scores (r = 0.10; *p* = 0.03).

Regarding psychological well-being, this variable showed a correlation with the MPAM-R score (r = 0.15; *p* < 0.001), as well as with intrinsic motivation (r = 0.23; *p* < 0.001). More specifically, psychological well-being was weakly related to the enjoyment (r = 0.22; *p* < 0.001), competence (r = 0.23; *p* < 0.001), social (r = −0.11; *p* = 0.02), and fitness (r = 0.25; *p* < 0.001) subscales of extrinsic motivation (Table 5).

In terms of the psychological well-being subscales, all of them were correlated to intrinsic motivation as well as the enjoyment and competence subscales, except for positive relations, which was only correlated to the enjoyment subscale (r = 0.10; *p* = 0.04). Regarding extrinsic motivation, it was noteworthy that only the autonomy subscale showed a negative correlation (r = −0.14; *p* < 0.001), and the subscale for purpose in life was positively correlated to this type of motivation (r = 0.18; *p* < 0.001). A remarkable fact was that the fitness subscale of extrinsic motivation was correlated to all the psychological well-being subscales except for autonomy (r = 0.09; *p* = 0.06), but this subscale showed a negative correlation with the appearance subscale (r = −0.18; *p* < 0.001).

### 3.5. Multiple Regression Analysis

In order to achieve the aims of this research, a three stepwise regression analysis was carried out for each dependent variable (physical activity, psychological well-being, and motivation). The collinearity of the variables was also verified using the collinearity statistics.

Firstly, the analysis of the IPAQ Level was carried out, adding independent variables to those with which physical exercise was correlated. The resulting model included the variables of intrinsic motivation, BMI, personal growth—all through a direct relationship with the dependent variable—and gender, where being a man determined a higher level of physical activity. This model explained 15.5% of the variability of the IPAQ Level (See Table 6).

Using the same approach, the regression analysis of the Ryff Psychological Well-Being Scales resulted in a model that explained 14.2% of the variability of psychological well-being. This model brought together the scores of intrinsic motivation and age in a positive way and also included the score for the social subscale belonging to the MPAM-R in an inverse way. Likewise, being a woman determined higher scores in psychological well-being. On the other hand, in the male group, this formed model explained 25.9% of the variance of the Ryff Psychological Well-Being Scales (See Table 6).

Finally, the analysis of the intrinsic motivation through the variables with which it showed a correlation resulted in a model that included vigorous-intensity activities, purpose in life, and gender, showing that being a man resulted in a greater intrinsic motivation. Therefore, this model explained the 19.8% variability in intrinsic motivation. In the male group, these variables positively determined intrinsic motivation, although this model explained 22.3% of its variability (See Table 6).

## 4. Discussion

The aim of this study was to analyze the relationship between physical exercise, motivation, and psychological well-being in young adults. In general, the data obtained reflect a profile of a young adult with a university education, with a higher percentage of women. Both men and women mostly had a BMI within a normal range and were physically active, with a higher level of physical activity among men. The mean scores showed moderate levels of both psychological well-being and motivation, obtaining higher scores at the level of intrinsic motivation. In these last two variables, it should be noted that the men showed higher scores related to high levels of activity.

This research has shown that performing physical activity has a direct relationship with the psychological well-being of the people who practice it. However, one of the main contributions of this work is reflected in the role of motivation in carrying out physical activity. Thus, although the importance of self-determined behaviors in exercise has been observed, this work also highlights the possible role that extrinsic motivation may have in starting physical activity and how it may have a certain importance when physical activity levels are high.

Therefore, responding to the aim of the study, it was possible to observe a correlation between physical activity and the psychological well-being of the participants. Furthermore, when analyzing the data according to the level of physical activity, higher mean scores were observed for a high level of activity. These data agree with the results of studies such as that of Marfil–Carmona et al. [47], in which participants showed greater psychological well-being when their physical activity was higher. These authors reported that this is mainly due to the fact that physical exercise promotes self-care and keeps people away from risk factors for their health. Moreover, in works such as that of Grasdalsmoen et al. [48] it has been observed that the frequency of physical exercise is negatively associated with depressive symptoms since physical activity favours the release of endorphins and maintains mitochondrial function. Furthermore, as An et al. [49] and Morgan et al. [50] state, physical activity promotes greater life satisfaction as it improves the general state of health and increases the individual’s self-efficacy, which directly influences their psychological well-being. When comparing the level of exercise between men and women, it was observed that, although both genders had a high and medium level of activity, men showed significantly higher levels of physical activity. Men tend to understand physical activity as a challenge, and their purpose is usually aimed at personal growth, which makes them have a better self-image and self-knowledge that is reflected in their greater psychological well-being [41,47]. However, it has been observed that the influence of the media results in them being unable to reach certain ideals of beauty and to feel greater dissatisfaction [47], which may fit in with the moderate scores found at the level of psychological well-being.

On the other hand, the data obtained indicated the existence of a correlation between motivation and physical activity so that at high levels of physical exercise, higher scores were obtained on the level of motivation. More specifically, significant differences were observed at the level of intrinsic motivation between the groups with different levels of physical activity, while at the level of extrinsic motivation these differences were only observed between the group with high levels of activity and the rest. These data agree with various studies [41,51,52,53] that show that physical activity is related to the most self-determined types of motivation, in addition to showing a slight influence of extrinsic motivation when the activity is vigorous. González–Hernández et al. [54] report that people perform physical exercise because they are motivated by internal elements, such as feeling pleasure, escaping or feeling competent, and by external elements, such as being strong and, above all, interacting with other people and having fun. Following this same line, the work of Tao et al. [36], points out the importance of extrinsic motivation to start exercising, with support from family and friends being a key element—as seen in the data of this study. Nevertheless, they understand that intrinsic motivation has a fundamental role in maintaining physical activity, and perhaps for this reason it is more strongly related to higher levels of physical activity [36]. Therefore, the results found in this research reflect the basis of the SDT, since they show that the development of self-determined behaviors (intrinsic motivation) favors adherence to physical activity and allows greater satisfaction with it. However, the data presented show that external pressure to behave in a certain way or achieve certain goals (extrinsic motivation) also seems to play a key role in starting physical activity [33,36,37,38,39,40].

In relation to these data, the linear regression model showed that intrinsic motivation was the most determining factor in predicting the level of physical activity, acquiring greater weight among men. This model agrees with other works [41,47,55] that discovered that men exercise to improve their self-image, with the aim of fulfilling a life purpose and overcoming challenges, which are characteristic elements of intrinsic motivation. However, women are motivated by factors such as improving their physical condition, losing weight, or reducing body fat, basing their motivation on elements that are external to the person [47,55]. In addition, with the data obtained from the multiple regression analysis, it was found that the intrinsic motivation model was influenced by vigorous activity in both men and women, indicating the feedback between the increase in intrinsic motivation and the level of activity. This finding coincides with the results of Sevil et al. [56], who point out that those with a higher level of activity show greater intrinsic motivation than people with a low level of activity, who have greater extrinsic motivation. This leads us to think that once people are capable of carrying out high-intensity physical activity, those skills that determine extrinsic motivation remain in the background, giving way to those related to intrinsic motivation [36,56]. Therefore, these data once again reinforce the idea exposed in the SDT, which suggests that acting of one’s own free will and for fun will favor adherence to physical activity, leaving aside those behaviors that seek external reinforcement and that involve less of the person in the physical activity [34,37,38,39,40,41]. However, as previously mentioned, certain works [41,51,52] report that extrinsic motivation may be present to a small extent in high-intensity activities, with factors such as being in shape or appearance having a certain importance.

Finally, as was the case with physical activity, intrinsic motivation was correlated with psychological well-being, given that that the greater the motivation, the greater their well-being. These data agree with the results of Martín–Albo et al. [57] and Marfil–Carmona et al. [47], who show that people who have a higher intrinsic motivation tend to have a better self-perception, which produces an increase in their psychological well-being by feeling more competent and more satisfied. In addition, these data are reinforced by the relationship observed between the enjoyment subscale, a determinant of intrinsic motivation, and psychological well-being in all its areas. Thus, enjoyment seems to be the intrinsic motivational factor that most influences the well-being of people with a moderate–high level of physical activity. These results coincide with those of a study carried out by de Vries, van Hooff, Geurts, & Kompier [58], which, in addition to demonstrating that enjoyment is closely linked to adherence to physical exercise, showed a clear relationship between those people who had a greater enjoyment when performing physical activity and their psychological well-being.

On the contrary, extrinsic motivation did not show a correlation with psychological well-being, also reflecting a negative relationship with the autonomy subscale within this variable, which may suggest that those people who depend more on external factors when exercising have less capacity to lead their lives autonomously. These data could be explained through the SDT. As reflected in this theory, the satisfaction of needs such as competence and autonomy imply greater intrinsic motivation and self-determination, and this leads to greater psychological well-being [33,36,37,38,39,40]. Moreover, these results concur with the study by Weman–Josefsson et al. [53], where it is shown that the extrinsic part of the motivation for exercise is reduced by providing support for the growth of autonomy. Furthermore, this autonomy was negatively related to elements of extrinsic motivation, such as concern for appearance or social relationships. This might indicate that those people who are more aware of their physique and appearance are less capable of leading their lives autonomously, which coincides with the results obtained by Miquelon et al. [59], who state that people who show greater dependence on their physical activity are less autonomous in their daily lives. In the same way, when observing the data from the linear regression model, this negative influence of the social aspect of motivation on psychological well-being in both men and women is reinforced so that it seems that when a motivation dependent on the social environment predominates, psychological well-being is impaired.

The study presents a series of limitations, which might condition the results found and therefore the conclusions drawn from it. First, the type of sampling makes it difficult to generalize the results. Second, self-administered surveys present a social desirability bias that makes subjects say what they are expected to answer or what is accepted by society in general and therefore hide the truth. Third, no objective measures of physical activity were collected; this information was solely obtained through a survey. Fourth, the availability of online and free access questionnaires makes it impossible for researchers to recognize whether the same subject answers the survey on more than one occasion, which could affect the data collected and the results found. Lastly, no causal relationships can be described due to the study design.

Therefore, it is necessary to carry out controlled and randomized experimental studies that investigate the true effects of physical exercise on the psychological well-being of those who practice it in the short, medium, and long term. In addition, the results found in this study provide relevant information to help health professionals work on motivation in order to achieve adherence to physical activity and improve the psychological well-being of their patients. Therefore, it would be interesting to develop recommendations and action plans that take into account that, in the initial stages, physical activity is influenced by social reinforcement, and, in later stages, self-determined behaviors will be the key factors for the consolidation of physical work. In addition, the data show that it is necessary to adopt different approaches according to gender, since the reasons for performing physical activity are different according to it. Finally, health professionals work with people who, due to different pathologies or health situations, do not have a good psychological well-being so that, according to what is exposed in this research, introducing physical activity could be an important factor in helping them to feel better.

## 5. Conclusions

The results of this study showed significantly higher scores on psychological well-being for those with a high level of physical activity compared to those with a lower level of activity. Men showed higher levels of physical activity and well-being, and self-knowledge and the approach to activity as a way of feeling competent could play an important role.

On the other hand, motivation was shown to be positively correlated with the different types of physical activity, with intrinsic motivation being the most important factor for starting and maintaining physical activity. This motivation was more present among men, for whom enjoyment and competition were key factors for physical activity, while in women, elements such as physical appearance or social relationships better defined the motivations for physical exercise. However, extrinsic motivation had a negative relationship with autonomy, which may suggest that those people who depend more on external factors when doing physical exercise have a lower capacity to lead their lives autonomously.

To sum up, it can be affirmed that exercise and interventions aimed at increasing motivation to do exercise could be a useful tool for improving the psychological well-being of the young adult population.

## Figures and Tables

**Table 1:** Demographic characteristics of the study sample.

Variable		*n*	%
Gender	Female	330	67.48
	Male	159	32.52
Educational level	Primary Education	22	4.5
	Lower Secondary Education	186	38.04
	Upper Secondary Education	280	57.26
	No Education	1	0.2
BMI	Underweight	24	4.91
	Healthy	341	69.73
	Overweight	91	18.61
	Obese	33	6.75
Exercise	Yes	273	55.83
	No	216	44.2
People influencing exercise	Friends	239	48.9
	Family	168	34.4
	Partner	62	12.7
	Coworkers	20	4.1
**Variable**	$\bar{X}$	**SD**	
Age	22.42	3.27	
BMI	23.63	4.56	

**Table 2:** Outcome measures of the study sample.

Variable		*n*	%
IPAQ	Low level	99	20.25
	Moderate level	174	35.58
	High level	216	44.17
**Variable**	$\bar{X}$	**SD**	
IPAQ	3701.39	3954.06	
Ryff Psychological Well-Being	4.47	0.69	
MPAM-R	3.39	0.76	

**Table 3:** Differences according to level of physical activity.

Variable	Overall	High Level	Moderate Level	Low Level	Z	*p*	Post Hoc Test
	*n* = 489	*n* = 216	*n* = 174	*n* = 99			
	Mean ± SD	Mean ± SD	Mean ± SD	Mean ± SD			
Age	22.42 ± 3.27	22.33 ± 3.26	22.72 ± 3.37	22.12 ± 3.13	1.21	0.3	
BMI	23.63 ± 4.56	24.18 ± 5.22	23.17 ± 3.81	23.26 ± 4.09	2.83	0.06	
IPAQ							
Walking	1491.31 ± 1859.72	2560.07 ± 2302.77	936.80 ± 599.30	134.07 ± 173.78	97.31	<0.001 ***	HL > LL
							HL > ML
							ML > LL
Moderate-intensity activities	790.72 ± 1386.99	1541.06 ± 1781.85	302.18 ± 434.56	12.24 ± 30.64	75.77	<0.001 ***	HL > LL
							HL > ML
Vigorous-intensity activities	1419.29 ± 2104.28	2849.96 ± 2453.95	445.98 ± 597.75	8.48 ± 60.01	143.99	<0.001 ***	HL > LL
							HL > ML
Ryff	4.47 ± 0.69	4.56 ± 0.68	4.43 ± 0.65	4.29 ± 0.73	11.73	<0.001 ***	DL > LL
Self-acceptance	4.26 ± 0.93	4.35 ± 0.94	4.28 ± 0.86	4.06 ± 1.02	7.76	0.021 *	
Positive relations	4.58 ± 1.03	4.65 ± 0.99	4.54 ± 1.03	4.49 ± 1.11	1.66	0.437	
Autonomy	4.35 ± 0.83	4.40 ± 0.85	4.36 ± 0.78	4.22 ± 0.88	4.76	0.093	
Environmental mastery	4.33 ± 0.83	4.43 ± 0.78	4.34 ± 0.83	4.11 ± 0.87	9.83	0.007 *	
Purpose in life	4.80 ± 0.76	4.63 ± 0.95	4.44 ± 0.96	4.24 ± 1.03	11.33	0.003 *	
Personal growth	4.48 ± 0.98	4.90 ± 0.75	4.79 ± 0.73	4.60 ± 0.78	13.17	<0.001 ***	
MPAM-R	3.39 ± 0.76	3.66 ± 0.62	3.26 ± 0.72	3.03 ± 0.89	30.82	<0.001 ***	HL > LL
							HL > ML
							ML > LL
Enjoyment	3.48 ± 1.06	3.87 ± 0.91	3.29 ± 1.00	2.96 ± 1.15	57.65	<0.001 ***	
Appearance	3.43 ± 0.96	3.55 ± 0.91	3.40 ± 0.95	3.21 ± 1.06	7.8	0.020 *	
Social	2.54 ± 0.97	2.76 ± 0.95	2.34 ± 0.94	2.41 ± 0.97	19.13	<0.001 ***	
Fitness	4.08 ± 0.85	4.33 ± 0.65	4.02 ± 0.85	3.65 ± 1.02	38.73	<0.001 ***	
Competence	3.43 ± 1.06	3.80 ± 0.92	3.24 ± 1.01	2.94 ± 1.13	53.32	<0.001 ***	
Intrinsic Motivation	3.45 ± 1.01	3.83 ± 0.87	3.27 ± 0.95	2.95 ± 1.10	35.15	<0.001 ***	HL > LL
							HL > ML
							ML > LL
Extrinsic Motivation	3.35 ± 0.71	3.55 ± 0.61	3.25 ± 0.67	3.09 ± 0.84	17.8	<0.001 ***	HL > LL
							HL > ML
		***n* (%)**	***n* (%)**	***n* (%)**	**X^2^**	***p***	
Gender							
Female		132 (40%)	122 (37%)	76 (23%)	8.44	0.02 *	
Male		84 (52.8%)	52 (14.5%)	23 (32.7%)			
Educational Level					9.76 **	0.09	
Primary Education		11 (50%)	7 (31.8%)	4 (18.2%)			
Lower Secondary Education		83 (44.6%)	55 (29.6%)	48 (25.8%)			
Upper Secondary Education		122 (43.6%)	111 (39.6%)	47 (16.8%)			
No Education		0 (0%)	1(100%)	0 (0%)			

SD: Standard deviation; Z: Kruskal–Wallis Test; X^2^: Chi-square test; HL: High Level; ML: Moderate Level; LL: Low level; ** Exact Fisher Test; * *p* < 0.05; *** *p* > 0.001.

**Table 4:** Differences according to gender.

Variable	Overall	Male	Female	Z	*p*
	*n* = 489	*n* = 159	*n* = 330		
	Mean ± SD	Mean ± SD	Mean ± SD		
Age	22.42 ± 3.27	22.56 ± 2.81	22.36 ± 3.48	−1.95	0.05
BMI	23.63 ± 4.56	24.21 ± 3.10	23.35 ± 5.09	−4.93	<0.001 ***
IPAQ	3701.39 ± 3954.06	4863.06 ± 4867.17	3141.56 ± 3293.54	−3.62	<0.001 ***
Walking	1491.31 ± 1859.72	1702.51 ± 2096.94	1389.55 ± 1728.12	−1.35	0.18
Moderate-intensity activities	790.72 ± 1386.99	1133.64 ± 1875.75	625.49 ± 1039.00	−3.23	0.001 ***
Vigorous-intensity activities	1419.29 ± 2104.28	2026.92 ± 2503.91	1126.52 ± 1814.57	−4.36	<0.001 ***
Ryff	4.47 ± 0.69	4.38 ± 0.74	4.51 ± 0.66	−1.85	0.06
MPAM-R	3.39 ± 0.76	3.50 ± 0.69	3.34 ± 0.78	−2.48	0.01 *
Intrinsic Motivation	3.45 ± 1.01	3.66 ± 0.92	3.35 ± 1.04	−2.99	<0.001 ***
Extrinsic Motivation	3.35 ± 0.71	3.40 ± 0.67	3.33 ± 0.73	−1.10	0.27

* *p* < 0.05; *** *p* < 0.001.

**Table 5:** Correlation analysis between studied variables.

Variable		IPAQ	MPAM-R	IM	Enjoy.	Comp.	EM	Social	Fitness	App.	Ryff	S-A	Pos. Rel.	Auton.	Environ.	Purp.life	Pers.Growth	Age	BMI
IPAQ	r	1.00																	
	*p*	-																	
MPAM-R	r	0.34	1.00																
	*p*	<0.001 ***	-																
IM	r	0.36	0.91	1.00															
	*p*	<0.001 ***	<0.001 ***	-															
Enjoy.	r	0.35	0.86	0.96	1.00														
	*p*	<0.001 ***	<0.001 ***	<0.001 ***	-														
Comp.	r	0.35	0.88	0.96	0.83	1.00													
	*p*	<0.001 ***	<0.001 ***	<0.001 ***	<0.001 ***	-													
EM	r	0.27	0.92	0.68	0.63	0.67	1.00												
	*p*	<0.001 ***	<0.001 ***	<0.001 ***	<0.001 ***	<0.001 ***	-												
Social	r	0.19	0.70	0.57	0.56	0.53	0.70	1.00											
	*p*	<0.001 ***	<0.001 ***	<0.001 ***	<0.001 ***	<0.001 ***	<0.001 ***	-											
Fitness	r	0.30	0.79	0.66	0.61	0.66	0.78	0.28	1.00										
	*p*	<0.001 ***	<0.001 ***	<0.001 ***	<0.001 ***	<0.001 ***	<0.001 ***	<0.001 ***	-										
App.	r	0.13	0.64	0.34	0.28	0.36	0.81	0.29	0.57	1.00									
	*p*	0.003 **	<0.001 ***	<0.001 ***	<0.001 ***	<0.001 ***	<0.001 ***	<0.001 ***	<0.001 ***	-									
Ryff	r	0.10	0.15	0.23	0.22	0.23	0.03	−0.11	0.25	−0.04	1.00								
	*p*	0.03 *	0.001 **	<0.001 ***	<0.001 ***	<0.001 ***	0.45	0.02 *	<0.001 ***	0.41	-								
S-A	r	0.78	0.15	0.22	0.20	0.21	0.04	−0.06	0.19	−0.05	0.84	1.00							
	*p*	0.08	0.001**	<0.001 ***	<0.001 ***	<0.001 ***	0.43	0.18	<0.001 ***	0.30	<0.001 ***	-							
Pos.Rel.	r	0.03	0.04	0.08	0.10	0.06	−0.02	−0.06	0.11	−0.06	0.67	0.44	1.00						
	*p*	0.51	0.43	0.06	0.04 *	0.16	0.66	0.21	0.02 *	0.21	<0.001 ***	<0.001 ***	-						
Auton.	r	0.06	−0.01	0.11	0.10	0.11	−0.14	−0.20	0.09	−0.18	0.71	0.52	0.40	1.00					
	*p*	0.19	0.82	0.02 *	0.02 *	0.02 *	0.002 *	<0.001 ***	0.06	<0.001 ***	<0.001 ***	<0.001 ***	<0.001 ***	-					
Environ.	r	0.08	0.13	0.20	0.18	0.20	0.04	−0.08	0.20	−0.01	0.87	0.75	0.42	0.56	1.00				
<0.001 ***	*p*	0.07	0.005 **	<0.001 ***	<0.001 ***	<0.001 ***	0.35	0.07	<0.001 ***	0.87	<0.001 ***	<0.001 ***	<0.001 ***	<0.001 ***	-				
Purp.life	r	0.11	0.25	0.29	0.26	0.30	0.18	0.03	0.30	0.09	0.84	0.73	0.39	0.48	0.78	1.00			
	*p*	0.01 *	<0.001 ***	<0.001 ***	<0.001 ***	<0.001 ***	<0.001 ***	0.54	<0.001 ***	0.04*	<0.001 ***	<0.001 ***	<0.001 ***	<0.001 ***	<0.001 ***	-			
Pers.Growth	r	0.11	0.12	0.16	0.16	0.17	0.06	−0.18	0.32	0.33	0.67	0.44	0.39	0.40	0.51	0.52	1.00		
	*p*	0.01 *	0.01	<0.001 ***	<0.001 ***	<0.001 ***	0.18	<0.001 ***	<0.001 ***	0.47	<0.001 ***	<0.001 ***	<0.001 ***	<0.001 ***	<0.001 ***	<0.001 ***	-		
Age	r	0.01	−0.05	−0.03	−0.04	−0.02	−0.05	−0.14	0.05	−0.02	0.12	0.08	0.01	0.14	0.16	0.09	0.09	1.00	
	*p*	0.97	0.28	0.45	0.35	0.63	0.25	0.003 *	0.28	0.64	0.01 *	0.07	0.83	0.002*	<.001***	0.06	0.05	-	
BMI	r	0.10	−0.03	−0.07	−0.07	−0.06	0.03	−0.04	0.01	0.07	−0.01	−0.04	−0.02	0.09	0.01	−0.03	−0.01	0.18	1.00
	*p*	0.03 *	0.54	0.13	0.15	0.20	0.56	0.34	0.83	0.12	0.98	0.42	0.70	0.06	0.79	0.46	0.91	<0.001 ***	-

* *p* < 0.05; ** *p* < 0.01; *** *p* < 0.001; r: Spearman Correlation Coefficient; IM: Intrinsic Motivation; Enjoy.: Enjoyment; Comp.: Competence; EM: Extrinsic Motivation; App.: Appearance; S-A: Self-acceptance; Pos.Rel.: Positive Relations; Auton.: Autonomy; Environ.: Environmental Mastery; Purp.life: Purpose of Life; Pers.Growth: Personal Growth.

**Table 6:** Linear regression analysis of physical activity, psychological well-being, and intrinsic motivation.

	B	Standard Error	β	Sig.	95% CI for B		R^2^
					Lower Limit	Upper Limit	
Model: IPAQ Level							0.16
Intrinsic Motivation	0.25	0.03	0.33	0.00	0.19	0.32	
BMI	0.02	0.01	0.12	0.005	0.01	0.03	
Personal Growth	0.11	0.04	0.11	0.009	0.03	0.20	
Gender	0.15	0.07	0.09	0.033	0.01	0.29	
Model: IPAQ Level (Female)							0.16
MPAM-R	0.32	0.37	0.32	0.00	0.22	0.42	
Personal Growth	0.20	0.05	0.18	0.00	0.09	0.31	
BMI	0.02	0.06	0.13	0.01	0.01	0.04	
Model: IPAQ Level (Male)							0.14
Intrinsic Motivation	0.29	0.06	0.37	0.00	0.18	0.41	
Model: Ryff							0.14
Intrinsic Motivation	0.28	0.04	0.42	0.00	0.22	0.35	
Social (MPAM-R)	−0.22	0.04	−0.31	0.00	−0.29	−0.15	
Age	0.02	0.01	0.10	0.02	0.003	0.04	
Gender	−0.14	0.06	−0.10	0.02	−0.27	−0.02	
Model: Ryff (Female)							0.08
Intrinsic Motivation	0.22	0.04	0.34	0.00	0.13	0.30	
Social (MPAM-R)	−0.19	0.05	−0.28	0.00	−0.28	−0.10	
Model: Ryff (Male)							0.26
Intrinsic Motivation	0.22	0.04	0.34	0.00	0.13	0.30	
Social (MPAM-R)	−0.19	0.05	−0.28	0.00	−0.28	−0.10	
Model: Intrinsic Motivation							0.20
Vigorous-intensity activities	0.001	0.001	0.32	0.00	0.00	0.00	
Purpose in life	0.27	0.04	0.26	0.00	0.19	0.35	
Gender	0.22	0.09	0.10	0.01	0.05	0.40	
Model: Intrinsic Motivation (Female)							0.18
Vigorous-intensity activities	0.001	0.001	0.33	0.00	0.00	0.00	
Purpose in life	0.24	0.06	0.22	0.00	0.13	0.35	
Model: Intrinsic Motivation (Male)							0.22
Purpose in life	0.31	0.06	0.34	0.00	0.18	0.43	
Vigorous-intensity activities	0.001	0.001	0.31	0.00	0.00	0.00	

CI: Confidence Interval.

## Data Availability

Not applicable.
